# Tracking bacterial virulence: global modulators as indicators

**DOI:** 10.1038/srep25973

**Published:** 2016-05-12

**Authors:** Alejandro Prieto, Imanol Urcola, Jorge Blanco, Ghizlane Dahbi, Maite Muniesa, Pablo Quirós, Linda Falgenhauer, Trinad Chakraborty, Mário Hüttener, Antonio Juárez

**Affiliations:** 1 Departament de Microbiologia, Facultat de Biologia, Universitat de Barcelona, Avda, Diagonal 643, 08028, Barcelona, Spain; 2 Institut de Bioenginyeria de Catalunya (IBEC), Baldiri Reixach 15-21, 08028, Barcelona, Spain; 3 Laboratorio de Referencia de E. coli (LREC), Departamento de Microbioloxía e Parasitoloxía, Facultade de Veterinaria, Universidade de Santiago de Compostela, 27002, Lugo, Spain; 4 Institute of Medical Microbiology, Justus-Liebig University, Schubertstrasse 81, 35392 Giessen, Germany and German Center for Infection Research DZIF, Partner site Giessen-Marburg-Langen, Campus Giessen, Germany

## Abstract

The genomes of Gram-negative bacteria encode paralogues and/or orthologues of global modulators. The nucleoid-associated H-NS and Hha proteins are an example: several enterobacteria such as *Escherichia coli* or *Salmonella* harbor H-NS, Hha and their corresponding paralogues, StpA and YdgT proteins, respectively. Remarkably, the genome of the pathogenic enteroaggregative *E. coli* strain 042 encodes, in addition to the *hha* and *ydgT* genes, two additional *hha* paralogues, *hha2* and *hha3*. We show in this report that there exists a strong correlation between the presence of these paralogues and the virulence phenotype of several *E. coli* strains. *hha2* and *hha3* predominate in some groups of intestinal pathogenic *E. coli* strains (enteroaggregative and shiga toxin-producing isolates), as well as in the widely distributed extraintestinal ST131 isolates. Because of the relationship between the presence of *hha2*/*hha3* and some virulence factors, we have been able to provide evidence for Hha2/Hha3 modulating the expression of the antigen 43 pathogenic determinants. We show that tracking global modulators or their paralogues/orthologues can be a new strategy to identify bacterial pathogenic clones and propose PCR amplification of *hha2* and *hha3* as a virulence indicator in environmental and clinical *E. coli* isolates.

Epidemiology of bacterial infections is in some instances understood because of the distribution of virulence genes in clinical isolates^1^. *Eschericha coli* virulent strains are a good example for that. This microorganism represents an outstanding example of genetic plasticity^2^ and of how the mechanisms driving horizontal gene transfer (HGT) impact its ability to colonize several niches, including human organs and tissues. Whereas several *E. coli* isolates are non-pathogenic and some of them belong to the human intestinal flora, many other strains express virulence determinants, which allow them to proliferate and cause disease. Pathogenic *E. coli* isolates are classified in pathotypes, which are defined by a combination of virulence factors, phenotype and clinical association^3^. However, the distribution of virulence factors is not strictly associated to each pathotype. A well-known example is *E. coli* strain O104:H4 that caused a large outbreak of bloody diarrhea with a high prevalence of associated hemolytic–uremic syndrome (HUS) in Germany in 2011^4^. This newly emerged strain caused the highest frequency of HUS and death ever recorded. The O104:H4 outbreak strain was classified as an enteroaggregative *E. coli* (EAEC) because of its pattern of adherence to cultured cells and the presence of a plasmid (pAA) that encoded the fimbriae that mediate this type of adherence^5^. In contrast to typical EAEC strains, the outbreak strain contains a prophage encoding the Shiga toxin^6^, which is a well-studied virulence determinant usually expressed by a different *E. coli* pathotype, enterohemorrhagic *E. coli* (EHEC). Remarkably, the strain contains an unusual combination of genes that accounts for its pathogenicity and its extensive antibiotic resistance profile against a variety of beta-lactams^5^,^7^. Pathogenic bacterial isolates containing different combinations of genes justify that identification of specific pathogenic lineages may require a complex analysis of the presence of a large set of virulence traits.

In enteric bacteria, regulation of virulence determinants is dependent upon, among other global regulators, the nucleoid-associated protein H-NS. This protein is widespread in Gram-negative bacteria and has been best studied in *E. coli* and related genera. H-NS plays a dual role, both as an architectural protein that contributes to the nucleoid structure and as a global modulator of gene expression (for a review see^8^). The *E. coli hns* gene encodes a 137-amino-acid protein with a molecular mass of 15.4 kDa. H-NS binds to DNA in a non-sequence-specific manner, but with a preference for intrinsically curved AT-rich regions. Genome-wide ChIP- and microarray studies have identified the set of genes that are modulated by H-NS^9^. They are mainly located in HGT DNA and include several virulence determinants^10^,^11^,^12^.

The Hha family includes a group of sequence-related low molecular mass proteins (about 8 kDa) involved in gene regulation in the enterobacteria. These proteins show structural mimicry to the H-NS N-terminal domain and interact with this protein to modulate gene expression (as reviewed in^13^). The *hha* gene can be present in one or more copies per chromosome or in plasmids in members of the *Enterobacteriaceae* family, but not in other genera such as *Vibrio* or *Aeromonas*, which also express proteins of the H-NS family^14^. Studies on the mechanism of action of the Hha protein are based on the Hha-mediated down-regulation of the *hlyCABD* operon in *E. coli*, which encodes the toxin α-haemolysin. Instead of binding to specific regulatory sequences, Hha binds to H-NS, which in turn binds to specific regions of the *hly* operon^15^. Several Hha targets are HGT genes^10^,^16^, which include various virulence determinants from enteric bacteria, comodulated with H-NS^14^,^17^,^18^.

A general rule in several enterobacterial isolates as, for instance, *Salmonella* and *E. coli* strains, is the presence of both a paralogue of the *hns* and *hha* genes (the *stpA* and the *ydgT* genes respectively) in their genomes. In addition, orthologues of *hns* and *hha* are also encoded in several conjugative plasmids^19^. The role of H-NS and Hha paralogues is not yet well characterized. Both the StpA and YdgT proteins are overexpressed in mutants lacking either H-NS or Hha and, in these backgrounds, overexpression of the paralogues appears to attenuate the phenotype of either *hns* or *hha* mutants^14^,^20^. Other roles for both paralogues are not ruled out.

A recent genomic analysis performed by our group has shown that, unlike many other *E. coli* strains, the chromosome of the EAEC strain 042^21^ encodes four paralogues of the *hha* gene: *hha, ydgT* and the hitherto undescribed *hha2* and *hha3* alleles (Fig. 1). By studying their distribution among a large number of commensal and pathogenic *E. coli* strains, we provide in this report convincing evidence for the association of the presence of these allelles to highly virulent *E. coli* isolates. We also provide information about their biological role. We propose tracking alleles of global modulators as a new approach to identify and characterize pathogenic bacterial isolates.

## Results

### Identification of *hha* paralogues EC042_4516 and 4796 in the genome of the EAEC strain 042

**Figure 1:**
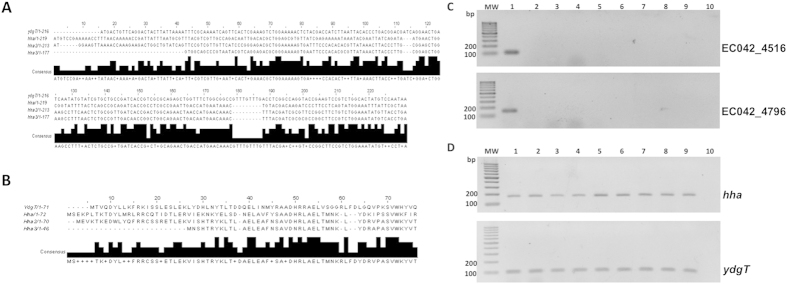
Alignment of nucleotide (**A**) and aminoacid (**B**) sequences of Hha, Hha2 and Hha3. PCR amplification of the *EC042_4516* (*hha2*) and *EC042_4796* (*hha3*) genes (**C**), and *hha, hha2, hha3* and *ydgT* genes (**D**) in strain 042 and different commensals *E. coli* strains. MW, molecular weight marker (GeneRuler 100 bp DNA Ladder, ThermoFisher Scientific); Lane 1, strain 042; lane 2, strain MG1655; lane 3, strain 5K; lane 4, strain XL1Blue; lane 5, strain BL21 (DE3); lane 6, strain DH5α; lane 7, strain ED1a; lane 8, strain ECO 01; lane 9, strain ECO 06; lane 10 negative PCR control.

*hha* paralogues EC042_4516 and EC042_4796 (from here on termed *hha2* and *hha3* respectively) were identified in the annotated genome of strain 042 by performing a Blast searching (http://www.uniprot.org/blast/) using the amino acid sequence of the Hha protein (Uniprot - D3GU89) as a template. Figure 1A,B show the nucleotide and aminoacid sequence alignment of Hha and putative Hha2 and Hha3 proteins. The *ydgT* gene was hitherto the unique chromosomally encoded *hha* paralogue described, and can be detected in all *E. coli* isolates. We decided to use PCR to assess the distribution of Hha, YdgT and putative Hha2 and Hha3 proteins in a large number of *E. coli* isolates. Taking into account the high degree of similarity of *hha, hha2* and *hha3* genes, we designed primers that would specifically amplify *hha2* and *hha3* but not *hha.* To do this we performed a search for specific regions within *hha2/hha3* paralogues by using the Primer Blast (http://www.ncbi.nlm.nih.gov/tools/primer-blast/). Specific primers to amplify *hha2* and *hha3* genes (4516 forward/reverse and 4796 forward/reverse (Supplementary Table 1 and Supplementary Fig. 1)) were selected. To confirm their specificity, we used strains 042 (positive control) and the already sequenced MG1655, DH5α, and BL21 *E. coli* strains (Table 1). In addition, the *E. coli* lab strain 5K and the commensal strain ED1a were also assayed. PCR analysis of the above-referred strains with the designed primers specific for *hha2* and *hha3* paralogues confirmed that they amplify these two *hha* alleles in strain 042, but not in the rest of *E. coli* strains analysed which, as expected, encode *hha* and *ydgT* (Fig. 1C,D).

### Distribution of *hha2* and *hha3* genes among *E. coli* strains belonging to different pathotypes

The fact that *hha2* and *hha3* were not detected in the different *E. coli* laboratory strains tested led us to hypothesize that these genes might be predominantly encoded in pathogenic *E. coli* isolates. To assess their presence in the different *E. coli* pathotypes, we used a LREC collection of 60 strains that includes: 16 enteroaggregative *E. coli* (EAEC), 21 Shiga toxin (verotoxin)-producing *E. coli* (*STEC/VTEC*), 3 enterotoxigenic *E. coli* (ETEC), 2 typical enteropathogenic *E. coli* (tEPEC), 2 enteroinvasive *E. coli* (EIEC), 1 adherent-invasive *E. coli* (AIEC), 14 extraintestinal pathogenic *E. coli* (ExPEC) and 1 commensal isolate (Supplementary Table 2). Presence of *hha2* and *hha3* genes could be unambiguously assessed by PCR analysis using the above-indicated specific primers for these genes (Supplementary Fig. 2A,B). Interestingly, 62.5% of all EAEC isolates harbored either *hha2* (62.5%) or *hha3* (50%) alleles (Table 2). Distribution of *hha2* and *hha3* genes between STEC/VTEC strains was lower than those observed among EAEC isolates (43% for *hha2* and 33% for *hha3*). With respect to ExPEC strains, the incidence of both paralogues is biased depending upon the sequence type: it is high among ST131 clone (67% for both genes) and low for the rest (38 and 13% for *hha2* and *hha3* respectively) (Table 2). It is important to point out here that all strains used in this study encode both *hha* and *ydgT* genes (Supplementary Fig. 3A,B).

Because of the high prevalence of *hha2* and *hha3* genes in the set of EAEC strains initially tested, we decided to perform a more comprehensive analysis of the presence of these paralogues in a larger number of EAEC isolates, and to try to correlate it with the virulence factors expressed by these isolates (Supplementary Table 3). As expected from the preliminary analysis with 16 EAEC strains (Table 2), the analysis of a total number of 56 EAEC strains confirmed the prevalence of both alleles. Allele *hha2* was present in 37 (66%) strains (in 10 of them alone, in the rest in combination with *hha3*). Allele *hha3* was present in 29 strains (52%) (in 2 of them alone, in the rest in combination with *hha2*). Both alleles together are present in 27 strains (48%). Remarkably, all three O104:H4 EAEC strains analysed showed one (2 strains *hha*2) or both (1 strain) alleles, and 5 of 6 O3:H2 EAEC strains were positive for one (*hha*2) or both alleles. In contrast, all five O86:H2 EAEC strains analysed did not show any of both paralogues.

The genes encoding the following virulence factors of EAEC were detected by PCR: Antiaggregation protein transporter (*aatA* gene), AAF/I fimbrial subunit (*aggA gene*), AAF/II fimbrial subunit (*aafA* gene), AAF/III fimbrial subunit (*agg3A* gene), transcriptional activator (*aggR* gene), aggregative heat-stable toxin 1 (EAST1) (*astA* gene), anti-aggregation protein (Dispersin) (*aap* gene), *Shigella* enterotoxin 1 mucinase (*set1A* gene), yersiniabactin (*irp2* gene), serine protease Pet (*pet* gene), cryptic ORF (*sf*h gene), secreted autotransporter toxin (*sat* gene), serine protease Pic (*pic* gene) and antigen 43 (*agn43* gene). The 14 virulence determinants studied are usually associated to EAEC strains, although *sat* is also associated to ExPEC strains and *astA* is associated to different pathotypes. From these genes, five (*aggA*, *astA*, *shf*, *sat* and *agn43*) were significantly associated with the presence of *hha2* and *hha*3 alleles (see Supplementary Table 3).

To complete the analysis we examined a collection of 25 *E. coli* isolates from German humans and companion animals comprising mainly ExPEC^22^ (Supplementary Table 4). *hha2* was present in 32% of the strains, and *hha3* in 56% of the strains (Table 3). Remarkably, these alleles predominate in ExPEC/EAEC strains, and are very infrequent in those isolates considered as intestinal flora (Table 3). We took advantage of the fact that these strains have been sequenced to correlate PCR data with genomic data (Supplementary Table 5). There exists a good correlation between the PCR analysis and the *in silico* detection of *hha2*/*hha3*.

### Distribution of *hha2* and *hha3* genes among the ECOR collection

We also decided to analyse the 72 members of the ECOR collection for the presence of *hha2*/*hha3*. The ECOR collection is a widely used set of 72 wild-type *E. coli* strains isolated between 1973 and 1983 from a variety of animal hosts and a variety of geographic locations^23^. The collection is thought to broadly represent genotypic variation in *E. coli*^24^. Although it was initially stated that none of the ECOR strains is pathogenic^25^, different reports have provided evidence for pathogenic *E. coli* being grouped among the ECOR strains on the basis of MLEE^26^. Furthermore, pathogenicity determinants for uropathogens, such as *pap, hly, kps* and *sfa*, are present in the genomes of some among of the ECOR strains^27^, though it is unclear whether or not these genes are active. Thus, 29 of 72 strains of ECOR collection showed the ExPEC status (with two or more of the following five virulence genes: *pap*, *sfa/foc*, *afa*, *aer* and *kps*)^28^ (Supplementary Table 6). PCR analysis of the distribution of *hha2* and *hha3* alleles showed that only 15% of the EcoR strains harbour any of them. Remarkably, both alleles were dectected in 4% of the strains only (Table 4).

### Distribution of *hha2* and *hha3* genes in environmental *E. coli* isolates

To obtain a broader view about the distribution of both *hha* paralogues in *E. coli*, we decided to investigate their presence in the genomes of environmentally isolated *E. coli* strains. To perform this study we used firstly a collection of 84 environmental *E. coli* isolates that were selected because they encode the *stx2* gene (Supplementary Table 7). These strains were isolated either from raw sewage samples of urban origin, mostly contaminated by human faecal wastes, or from wastewater samples from three different abattoirs (cattle, pig, and a mixed cattle, lamb, goat slaughterhouse). The strains were isolated from the water samples only on the basis of the *stx*_*2*_ presence. Samples were isolated as previously described^29^. Secondly, we isolated a further set of 88 *E. coli* strains from environmental samples, which, without further characterization, were tested for the absence of *stx* and the presence of *hha2* or *hha3*. In spite of their environmental origin, there was a significant bias in the distribution of the *hha* alleles in both sets of strains. *hha2* alone was present in 35 out of the 84 Stx-producing *E. coli* strains (41.6%). *hha3* alone was not present in any strain. Both alleles were present in six out of the 84 strains (7.1%) (Table 5). In contrast, when considering the 88 non-characterized Stx^−^ environmental isolates, *hha2* allele alone was present in ten strains (11.3%) (Significant differences versus Stx_2_^+^ strains (P = 0.000019)), *hha3* alone was present in two strains (2.3%) and the combination of both in nine strains (10.2%).

### Identification of *hha2* and *hha3* in the annotated *Escherichia coli* genomes

**Figure 2:**
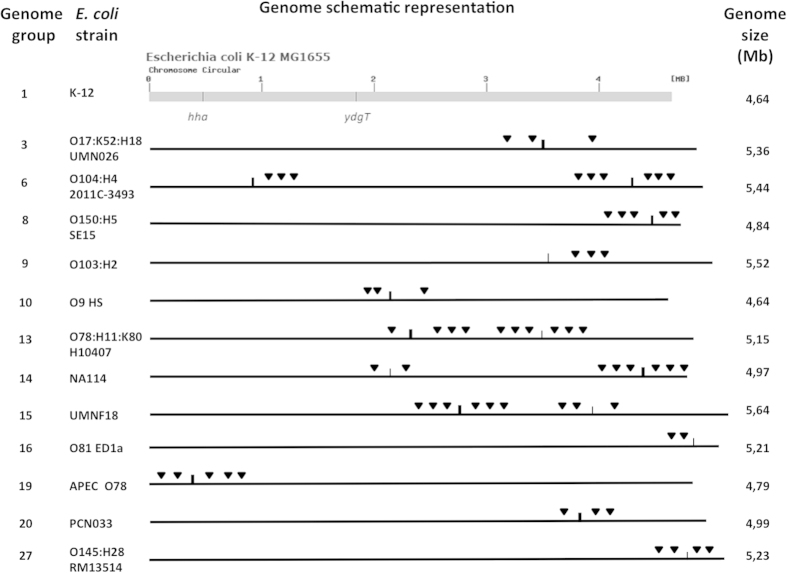
Map position of *hha2* and *hha3* genes in representatives of the different groups of NCBI annotated *E. coli* genomes. In the top is drawn the genome of strain MG1655 including positions of *hha* and *ydgT*, which are fairly conserved in almost all *E. coli* genomes analysed. Thin line means *hha2*; thick line means *hha3*. Triangles correspond to transposase-like genes flanking either *hha2* or *hha3*.

The annotated *E. coli* genomes in NCBI are grouped in 33 groups, each of which has a representative strain. We used the BLASTN (90% identity and 80% coverage) to detect *hha2* and *hha3* in 20 strains whose genome is complete. 16 strains correspond to representative strains of each group, and 4 to members of groups whose representative strain has not been completely sequenced. *hha2* and *hha3* homologues could be identified in twelve strains (Fig. 2). Representative strain of group 1 is MG1655. As expected, any of both genes could be detected in this strain (data not shown). We extended our analysis to the rest of the strains of this group whose genome is completely sequenced. None of them expressed these paralogues. Identification of *hha2* and *hha3* was possible mainly in pathogenic *E. coli* representatives, out of the commensal strains SE15, O9H5 and ED1a, but this latter contains a 3 bp deletion of the *hha2* homologue, which most likely results in function loss. We also mapped *hha, ydgT, hha2* and *hha3* genes in the corresponding genetic maps (Fig. 2). As expected from core genes, position of *hha* is similar in all genomes except that of strain O104:H4 (most likely this being due to a genomic rearrangement). This is also the case for *ydgT*. In contrast, *hha2* and *hha3* alleles map in different positions in the different strains that harbour them, and they are flanked by insertion elements sequences. As well, *hha2* and *hha3* are distributed randomly in the different groups.

### A regulatory role for the *hha2* and *hha3* gene products: modulation of the expression of the ag43 determinants of strain 042

**Figure 3:**
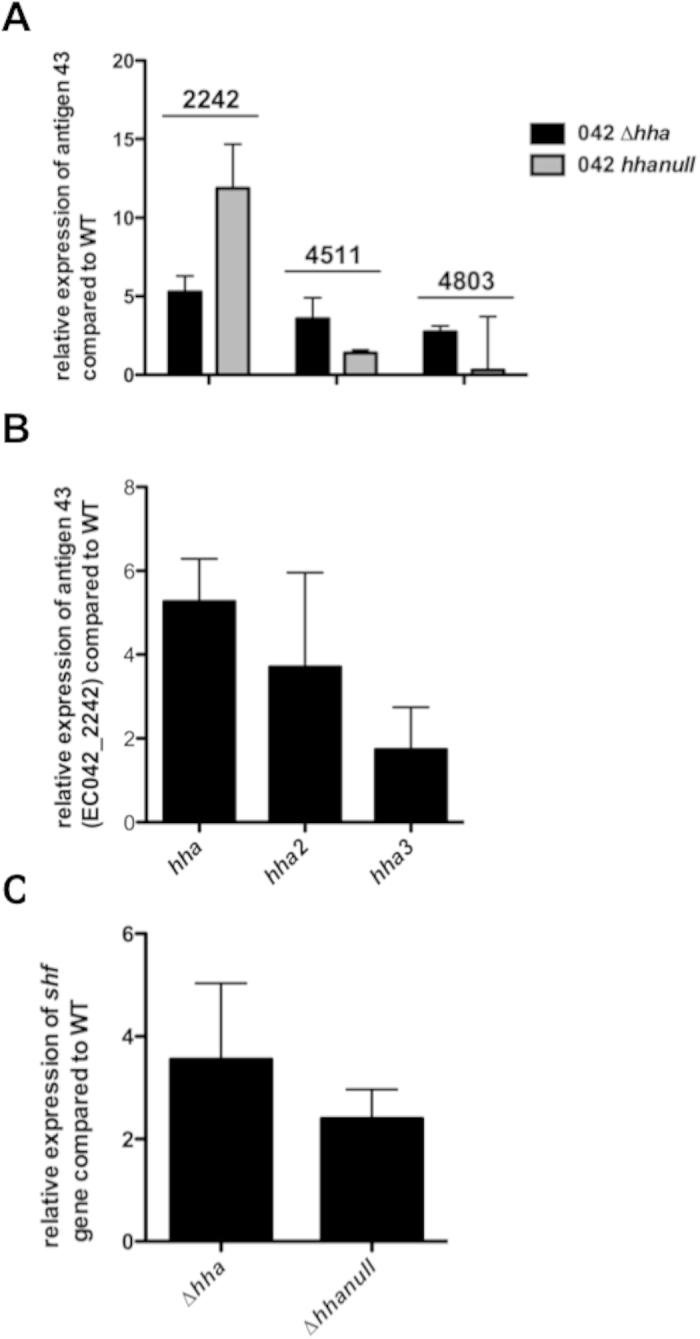
(**A**) Relative expression of the three antigen 43 determinants encoded in *E. coli* strain 042. Expression levels of the different *ag43* alleles in the *hha* and *hha* null mutants compared with those of the wt strain. (**B**) Expression of the antigen 43 (EC042_2242) in *hha*, *hha2* and *hha3* mutants compared with those of the wt strain. (**C**) Expression of the *sfh* gene in 042 *hha* and *hha* null strains, compared with that of the wt strain.

As commented above, the analysis of the presence of *hha2* and *hha3* genes in the set of EAEC strains analysed showed a correlation with the presence of, among others, the virulence factors *shf* and *ag43*. We decided to assess if any or both paralogues play a role modulating their expression. To evaluate this, we first constructed isogenic derivatives of strain 042 lacking either *hha*, *hha2, hha3* or all three paralogues simultaneously. Analysis of the 042 genome annotation showed that this strain contains three different copies of the *ag43* gene (*EC042_2242, 4511* and *4803*)^30^, but only one copy of the *shf* gene. We analysed the expression of the three *ag43* copies and the *shf* gene in the different genetic backgrounds. Samples were obtained from cultures grown at 37 °C and entering the stationary phase (OD_600_ 2.0). The analysis of the expression of the three different alleles of the *ag43* gene was performed by qRT-PCR (Fig. 3A). For one of the *ag43* alleles (*EC042 _2242*), all three *hha* paralogues modulate its expression. In contrast, expression of the other two is not significantly influenced by Hha or its paralogues (Fig. 3B). With respect to the *shf* gene, the results obtained show that the Hha protein represses its expression under the conditions tested (Fig. 3C). In this example, the other two *hha* paralogues do not appear to significantly participate in the modulation of *shf.*

### Comparative expression of *hha*, *hha2* and *hha3* alleles in strain 042

**Figure 4:**
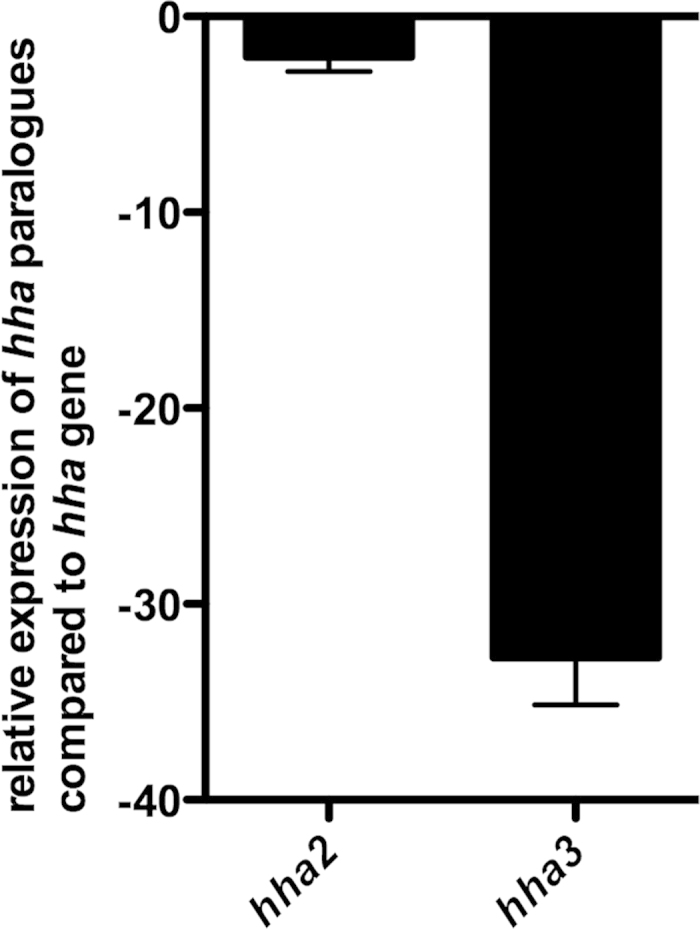
Expression of *hha2* and *hha3* compared to that of *hha* gene.

Expression of global modulators is a key aspect of their regulatory role. It is well known that proteins such as FIS show an increased expression in the exponential growth phase, other such as IHF are mainly expressed in the stationary growth phase^31^,^32^. Former studies showed that the Hha protein is reduced in salt-free LB medium^33^. We decided to compare expression of all three paralogues in strain 042. Samples were obtained from cultures grown at 37 °C to the early stationary phase (OD_600 nm_ 2,0). Expression of all three paralogues was assessed by qRT-PCR (Fig. 4). *hha*2 expression is slightly lower than *hha* expression. Remarkably, expression of *hha*3 is significantly lower than those of the other two paralogues.

## Discussion

Temporal and geographical surveillance of high-risk bacterial pathogenic clones is a relevant issue to prevent outbreaks. Identification of these clones is performed by a combination of both classical typing and subtyping protocols (i.e., detection of genes and/or gene products associated to virulence, serotyping, bacteriocin typing, antimicrobial testing and other phenotypic traits such as biochemical or enzymatic activities) with more recently developed genotyping approaches (i.e., pulse field electrophoresis, multilocus sequence typing or whole-genome sequencing (WGS)). WGS can be used for epidemiological applications (as reviewed in^34^), but also to define phenotypic characteristics (i.e., virulence) of a particular pathogen. Among these approaches, PCR has and is being used as a routinely tool to detect the distribution of virulence determinants that is frequently used to understand the epidemiology of bacterial infections^1^.

Due to the relevance of *E. coli* as an indicator of faecal contamination, there have been developed several methods for the detection of this microorganism in environmental as well as in food samples. In contrast to tedious and time-consuming classical methodologies such as the Multiple Tube Fermentation technique, approaches such as the PCR, are rapid, highly specific and sensitive^35^,^36^. Different genes, such as 16S rDNA, *EF-Tu*^37^, *uidA* or *yaiO*^38^ have been used to detect *E. coli* by PCR in environmental samples. For the detection of specific *E. coli* pathotypes, combinations of primers specific for virulence factors are designed^39^,^40^,^41^. Considering the high *E. coli* genetic plasticity and because of the impact of HGT shaping *E. coli* bacterial genome^2^, it is not surprising that isolates belonging to a specific *E. coli* pathotype may display unusual combinations of virulence factors. Even some of the hitherto considered commensal strains encode well-characterized virulence factors^39^,^42^,^43^,^44^. Hence, widely spread virulence markers different from classical virulence factors may help to identify pathogenic *E. coli* isolates.

Whereas several bacterial global modulators have been thoroughly studied in the recent past, this is not the case with their corresponding paralogues or orthologues. Plasmid-encoded orthologues of genes coding for nucleoid-associated proteins are not rare^19^ but again, their biological significance remains in many instances unclear. We show in this report that the study of paralogues or orthologues of global modulators may be of great relevance to better understand the biology of bacterial cells. In this context, the association of alleles of global modulators to specific *E. coli* pathotypes or highly virulent clones provides a new tool for the surveillance of pathogenic microorganisms. The presence of a global modulator must be necessarily associated to a set of genes rather than to a specific gene. These traits must in turn define a specific lifestyle of the isolate. Paralogues *hha2* and *hha3* match to this approach.

From the different sets of strains used for this study, *hha2* and *hha3* paralogues are mainly detected among EAEC, STEC/VTEC and ExPEC strains. With respect to this latter pathotype, it is remarkable that *hha2* and *hha3* are mainly associated to ST131 isolates. A correlation between *hha2, hha3* and intestinal pathogenic strains is further established when environmental strains are selected because of the presence of the *stx2* gene in their genomes. Whereas about 50% of the environmental strains encoding *stx2* have incorporated *hha2*/*hha3* alleles, their presence is only of about 20% when environmentally isolated *E. coli* strains lacking this virulence determinant are analysed. Analysis of the ECOR collection for the presence of the *hha* paralogues confirms the relationship between them and virulence. Within the ECOR strains, only of 15% encode *hha2* and 4% *hha3*. The presence of *hha2* and *hha3*, rather than being linked to a pathotype (i.e., EAEC), is a marker of a set of pathogenic isolates that includes clones causing some of the most severe *E. coli*-mediated infections (i.e., EAEC O104:H4 or the worldwide distributed ExPEC ST131 clone). PCR-mediated amplification of *hha2*/*hha3* in environmental or clinical samples is therefore a preliminary indicator of the presence of highly virulent *E. coli* strains which, in turn, can be further characterized by using primers specific of virulence genes that are associated to specific pathotypes.

A variable mapping position, their genomic context and their random distribution in the *E. coli* phylogenetic groups strongly suggest *hha2* and *hha3* spreading in *E. coli* strains by horizontal gene transfer mechanisms. Interestingly, these alleles are not detected in plasmids, which speaks for *hha2 hha3* spreading by mechanisms different to conjugation. It is reasonable to hypothesize that horizontal inheritance of *hha2* and *hha3* may be correlated with inheritance of other HGT-encoded genes, among others, virulence determinants.

In addition to the correlation between *hha2, hha3* and virulent *E. coli* isolates we provide in this work insight about the biological role of their gene products. *hha2* and *hha3 expression* is not significantly influenced by the growth conditions (i.e., exponential/stationary phase, high/low temperature, high/low osmolarity) (our unpublished results). By using as a model the EAEC strain 042, we show that, when growing in rich medium at 37 °C., modulation of the expression of one of the three copies of *ag43* that are encoded in this strain requires the participation of the *hha2* and *hha3* gene products and of the Hha protein itself. This was not the case for the *shf* gene, which is present in a single copy and is modulated by the *hha* gene product, but not by its paralogues. Hence, a likely hypothesis that may at least in part explain the occurrence of these paralogues is that amplification of virulence determinants such as *ag43* in some strains may positively select the amplification of the genes that encode for the global modulators that fine tune their expression. An intriguing aspect that deserves further research is the significant lower expression level of *hha3* compared to *hha* and *hha2*.

Analysis of virulence in pathogenic microorganisms usually takes into account the presence or absence of the corresponding virulence gene(s). The results we provide here clearly show that it is relevant to consider also the presence of specific alleles of global modulators. The increasing availability of the complete genomic sequences of several pathogenic strains will facilitate this analysis and will provide a more complete view of the complexity of the virulence regulons that some bacterial pathogens display.

## Methods

### Bacterial strains, plasmids and culture media

All bacterial strains used in this work are listed in Table 1. Cultures were normally grown in Luria Broth (LB) medium (10 g NaCl, 10 g tryptone and 5 g yeast extract per litre) with vigorous shaking at 200 rpm (Innova 3100, New Brunswick Scientific).

### Isolation of Stx^−^ *E. coli* strains from water samples

*E. coli* isolation was performed using the membrane filtration method according to previously standardized methods^45^. Briefly, serial decimal dilutions of urban wastewater and river water were filtered through 0.44-mm-pore-diameter membrane filters (EZ-Pak^®^ Membranes were placed on Chromocult^®^ coliform agar (Merck, Darmstadt, Germany) for selective *E. coli* growth, and incubated at 44 °C for 18 h. Blue colonies of each sample, corresponding to *E. coli*, were randomly subcultured and used for the study. Indol test was used to confirm that the isolates were *E. coli* and the absence of *stx* genes was confirmed by PCR.

### Genetic manipulations

All enzymes used to perform standard molecular and genetic procedures were used according to the manufacturer’s recommendations. To introduce plasmids in *E. coli*, bacterial cells were grown until a D.O_600 nm_ 0,6. Cells were then washed several times with glycerol 10%, and the respective plasmids were introduced by electroporation using an *Eppendorf* gene pulser (Electroporator 2510).

Mutant derivatives lacking alleles *hha, hha2* and *hha*3 in EAEC strain 042 were obtained by the λ Red recombinant method described by^46^. Briefly, the antibiotic-resistance cassette of kanamycin of plasmid pKD4 was amplified using oligonucleotides HhaP1/HhaP2, 4516P1/4526P2 and 4796P1/4796P2 for hha, *hha2* and *hha3* deletions, respectively (See Supplementary Table 1, for sequence). DNA templates were treated with DpnI (Thermo Scientific) following manufacturer recommendations, and, then, purified and electroporated to the competent cells. Mutants were selected on LB plates containing the appropriate selection marker (kanamycin in that case) and the successful deletion of the gene was confirmed by PCR using the primers KT (kanamycin resistance; Km^r^) in combination with specific primers located in the remaining gene sequence in the bacterial chromosome.

If necessary, the antibiotic resistance was eliminated by transforming the mutant strain with plasmid pCP20 and subsequent incubation at 42 °C for two or more passages as reported^47^. The pCP20 plasmid encodes the Flp recombinase that catalyses the recombination between the FRT sites flanking the kanamycin cassette^47^.

### Amplification of alleles *hha2* and *hha3* by PCR

To detect the prevalence of the different *hha* paralogues in the strains tested, we performed standard PCRs. One colony of each bacterial strain was diluted in 20 μl of sterile water and it was used as a template for the premix DreamTaq Green PCR Master Mix (Thermo Scientific), and, a final concentration of 0,4 μM of primers 4516FW–4516RV or 4796FW–4796RV, for paralogues *hha*2 and *hha*3, respectively (See supplementary Table 1, for sequence). PCRs were run using the following steps: initial denaturation for five minutes at 95 °C, following by 25 cycles of 95 °C denaturation temperature for 30 seconds, annealing temperature of 58 °C for 30 seconds and 30 seconds of 72 °C extension temperature followed by another ten minutes of final extension at 72 °C. The 25-cycle amplification was performed in a T100 thermal cycler (Biorad). For detection of PCR products, 10 μl of the amplified DNA was run on a 2% TAE 0,5×−agarose gel (Pronadisa), stained with ethidium bromide, and visualized under UV light using Gel Doc XR+ system (Biorad).

### Isolation of RNA

Total RNA was extracted from bacterial pellets using the RNeasy Mini kit (Qiagen) according to the manufacturer’s instruction. Potential traces of DNA were removed by digestion with DNase I (Turbo DNA-free, Ambion), according to the manufacturer’s instructions. RNA concentration and RNA quality were measured using a Nano- Drop 1000 (Thermo Fisher Scientific).

### qRT-PCR

Expression levels of *hha* paralogues and antigen 43 genes were analysed using real-time quantitative PCR. Briefly, 1 μg of total RNA isolated previously was reverse transcribed to generate cDNA using the High-capacity cDNA Reverse Transcription kit (Applied Biosystems) according to the manufacturer’s protocol. All samples within an experiment were reverse transcribed at the same time, the resulting cDNA diluted 1:100 in nuclease-free water and stored in aliquots at −80 °C until used. As a control, parallel samples were run in which reverse transcriptase was omitted from the reaction mixture.

Real-time PCR was carried out using SYBR green PCR master mix (Life Technologies) and the ABI Prism 7700 sequence detection system (Applied Biosystems). Specific oligonucleotides complementary to the genes of interest were designed using primer3 software. The primers were named 4516FW–4516RV or 4796FW–4796RV, for paralogues hha2 and hha3, respectively (see Supplementary Table 1). Relative quantification of gene expression of mutants versus wild type strain was performed using the comparative threshold cycle (CT) method. The relative amount of target cDNA was normalized using the *gapA* gene as an internal reference standard. Fold change values referring to relative expression of target genes in mutant strains versus wt strain were calculated by dividing the ΔCT (difference of Ct values between the target gene and the internal reference standard *gapA* gene) obtained for the different mutant strains versus wt strain.

### Statistical analysis

Proportions were compared between groups by use of the Fisher’s exact test. *P* < 0.05 was considered to denote significant differences.

## Additional Information

**How to cite this article**: Prieto, A. *et al.* Tracking bacterial virulence: global modulators as indicators. *Sci. Rep.* **6**, 25973; doi: 10.1038/srep25973 (2016).

## Supplementary Material

Supplementary Information

## Figures and Tables

**Table 1:** Bacterial strains and plasmids used in this study.

Bacterial strains	Description	Source or reference(s)
042	*E. coli* EAEC, Cm^r^ Sm^r^ Tc^r^	Prof. I. Henderson
042Hha	042Δ*hha* (EC042_0498)	This work
042Hha-2	042Δ*4516* (EC042_4516)	This work
042Hha-3	042Δ*4796* (EC042_4796)	This work
042Hhanull	042 Δ*0498*Δ*4516*Δ*4796*	This work
MG1655	*E. coli*, F^−^, *ilvG, rph1*	48
5KRif	*E. coli, F*^−^*, hsdR, hsdM, rpsL, thr, thi, leu, lac, spontaneus resistant to Rifampicin*	49
DH5α	*E. coli, fhuA2 lac(del)U169 phoA glnV44 Φ80′ lacZ(del)M15 gyrA96 recA1 relA1 endA1 thi-1 hsdR17*	50
XL1Blue	*E. coli, recA1 endA1 gyrA96 thi1 hsdR17 (rk− mk*+) *supE44 relA1 λ− lac− (F’ proA*+ *B* + *lacIq lacZΔM15 Tn10) Tcr*	51
Ed1a	*E. coli*, commensal strain	2
BL21DE3	*T7 polymerase upon IPTG induction*	52
Plasmids	Description	Source or reference(s)
pKD3	oriRγ, Cm^r^, Ap^r^	46
pKD4	oriRγ, Km^r^, Ap^r^	46
pKD46	oriR101, repA101 (ts), AraBp-gam-bet-exo	46
pCP20	λcI857 (ts), ts-rep (Recombinase FLP ts)	47
*E. coli c*ollections	Description	Source or reference(s)
60 strains (59 human and 1 avian) of different pathotypes (EAEC, ExPEC, STEC/VTEC, ETEC, tEPEC, EIEC, AIEC isolated in Spain, Germany, France, Denmark and USA)	See Supplementary Table 2	LREC collection (not published)
56 human EAEC strains isolated in Spain, Germany and Brazil	See Supplementary Table 3	LREC collection (not published)
25 ExPEC isolates	See Supplementary Table 4	22
ECOR collection	See Supplementary Table 5	24
84 *stx2*-positive environmental isolates	See Supplementary Table 6	53
88 *stx2*-negative environmental isolates		This work

**Table 2:** Distribution of *hha2* and *hha3* genes among *E. coli* strains belonging to different pathotypes.

Pathotype	Number of strains analysed	Number and percentage of strains containing only allele *hha-2*	Number and percentage of strains containing only allele *hha-3*	Number and percentage of strains containing both alleles *hha-2* and *hha-3*
EAEC	16	2 (12.5%)	0	8 (50%)
ExPEC	14	3 (21%)	1 (7%)	4 (29%)
ExPEC ST131	6	1 (17%)	1 (17%)	3 (50%)
ExPEC no-ST131	8	2 (25%)	0 (0%)	1 (13%)
STEC/VTEC	21	3 (14%)	1 (5%)	6 (29%)
ETEC	3	2 (67%)	1 (33%)	0 (0%)
tEPEC	2	0 (0%)	0 (0%)	1 (50%)
EIEC	2	0 (0%)	0 (0%)	0 (0%)
AIEC	1	0 (0%)	0 (0%)	0 (0%)

**Table 3:** Distribution of *hha2* and *hha3* genes among a collection of *E. coli* human and animal isolates.

Pathogenic category	Number of strains analysed	Number and percentage of strains containing only allele *hha-2*	Number and percentage of strains containing only allele *hha-3*	Number and percentage of strains containing both alleles *hha-2* and *hha-3*
ExPEC	14	1 (7.1%)	4 (28.5%)	4 (28.57%)
EAEC	2	0	1 (50%)	1 (50%)
Intestinal flora	5	0	2 (40%)	0
NA	4	1 (25%)	1 (25%)	1 (25%)

**Table 4:** Distribution of *hha2* and *hha3* genes among the ECOR collection.

Pathogenic cathegory	Number of strains analysed	Number and percentage of strains containing only allele *hha-2*	Number and percentage of strains containing only allele *hha-3*	Number and percentage of strains containing both alleles *hha-2* and *hha-3*
Commensal^a^	43	3 (6.9%)	4 (9.3%)	0 (0%)
ExPEC^b^	29	3 (10%)	4 (13.7%)	3 (10%)

^a^Commensal = non pathogenic *E. coli* strains without characteristic virulence genes of extraintestinal pathogenic *E. coli* (ExPEC) and intraintestinal pathogenic *E. coli* (InPEC).

^b^29 of 72 strains of ECOR collection showed the ExPEC status (with two or more of the following five virulence genes: *pap*, *sfa/foc*, *afa*, *aer* and *kps*)^28^.

**Table 5:** Distribution of *hha2* and *hha3* genes among *E. coli* strains isolated from environmental samples.

Stx production	Number of strains analysed	Number and percentage of strains containing only allele *hha-2*	Number and percentage of strains containing only allele *hha-3*	Number and percentage of strains containing both alleles *hha-2* and *hha-3*
+	84	35 (41.6%)	0 (0%)	6 (7.1%)
−	88	10 (11%)	2 (2%)	9 (10%)

Fisher’s exact test are shown where P < 0.05. Significant differences are indicated in bold (P = 0.000019).
