# Supplementary material for: Tracking bacterial virulence: global modulators as indicators
Source: Sci Rep. 2016 May 12;6:25973. doi: 10.1038/srep25973 (PMC4864382; doi:10.1038/srep25973)
Supplement: Supplementary Information [file srep25973-s1.doc]

**Tracking bacterial virulence: global modulators as indicators**

Alejandro Prieto^1^, Imanol Urcola^2^, Jorge Blanco^3^, Ghizlane Dahbi^3^, Maria Teresa Muniesa^1^, Pablo Quirós^1^, Linda Falgenhauer^4^, Trinad Chakraborty^4^, Mário Hüttener^2*^ and Antonio Juárez^1,2*^

^1^Departament de Microbiologia, Facultat de Biologia, Universitat de Barcelona, Avda. Diagonal 643, 08028. Barcelona, Spain.

^2^Institut de Bioenginyeria de Catalunya (IBEC), Baldiri Reixach 15-21, 08028. Barcelona, Spain*.*

^3^Laboratorio de Referencia de *E. coli* (LREC), Departamento de Microbioloxía e Parasitoloxía, Facultade de Veterinaria, Universidade de Santiago de Compostela, 27002. Lugo, Spain.

^4^Institute of Medical Microbiology, Justus-Liebig University, Schubertstrasse 81, 35392 Giessen, Germany and German Center for Infection Research DZIF, Partner site Giessen-Marburg-Langen, Campus Giessen.

^*^Corresponding authors. [ajuarez@ub.edu](mailto:ajuarez@ub.edu). Phone (+34) 934034624. Fax (+34) 934034627

Supplementary Table 1 – Oligonucleotides used in this study.

| **Oligonucleotide** | **Sequence (5’-3’)** |
| --- | --- |
| 4516 forward | 5’CTTACCCTTGCGGAGCTGGA 3’ |
| 4516 reverse | 5’TATTTCCAGACAGAAGCCGGAA 3’ |
| 4796 forward | 5’GAGACGCGGGAAAAGTGAAT 3’ |
| 4796 reverse | 5’CAGGTGACATATTTCCAGACGG 3’ |
| Hha P1 | 5’ATGTCCGAAAAACCTTTAACGAAAACCGATTATTTAATGCGTGTGTAGGCTGGAGCTGTCTTC 3’ |
| Hha P2 | 5’TTAGCGAATAAATTTCCATACTGAGGAAGGGATCTTGTCGTACATATGAATATCCTCCTTAGT 3’ |
| Hha P1 UP | 5’GTTAGAATTATTACAACCATGGG 3’ |
| hha forward | 5´GTTTACGTCGTTGCAGACAA3´ |
| hha reverse | 5´TTCCATACTGAGGAAGGGATCT3´ |
| ydgT forward | 5´ATGTATCGTGCTGCCGATCA3´ |
| ydgT reverse | 5´ATTGGACATAGTGCCAGACG3´ |
| 4516 P1 | 5’ATCACGTTAATTGCAGCATAAAGTGATGAGAGGCTAATGGAAGTGTAGCTGGAGCTGCTTC 3’ |
| 4516 P2 | 5’AACGCCCGGCACAATACCGCTGTGATTAACGAACGTTCAGGTCATATGAATATCCTCCTTAGT 3’ |
| 4516 P1 UP | 5’GTCTGAGCGGTAAACGTATC 3’ |
| 4796 P1 | 5’CGGAACACTAAGGCTGTATTGCGGGACACCTGGTAAGTCGCAGTGTAGGCTGGAGCTGCTTC 3’ |
| 4796 P2 | 5’GATAACACCGGAGTCATCGTAGCGCTGCTTCAATATTCAGGTCATATGAATATCCTCCTTAGT 3’ |
| 4796 P1 UP | 5’TCGTTCATGTATCAGAGTGC 3’ |
| UP378 | 5’GCGTTTTGACCATCTTCGT 3’ |
| LP378 | 5’ACAGGAGCAGTTTCAGACAG 3’ |
| KT | 5’CGGCCACAGTCGATGAATCC 3’ |

| Supplementary Table 2 - *E. coli* strains belonging to different pathotypes used in this study. | | | | | | |
| --- | --- | --- | --- | --- | --- | --- |
| **Pathotype** | **LREC code strain** | **Serotype** | **Sequence type** | **Phylogenetic group** | ***hha2*** | ***hha3*** |
| EAEC | 1 | O3:H2 |  |  | **+** | **+** |
| EAEC | 2 | O3:H2 |  |  | **+** | **+** |
| EAEC | 3 | O11:H- |  |  | **-** | **-** |
| EAEC | 4 | O17:H- |  |  | **+** | **+** |
| EAEC | 5 | O44:H18 |  |  | **+** | **+** |
| EAEC | 6 | ONT:HNT |  |  | **+** | **+** |
| EAEC | 7 | O3:H2 |  |  | **+** | **+** |
| EAEC | 8 | O15:H- |  |  | **-** | **-** |
| EAEC | 9 | O17:H18 |  |  | **+** | **-** |
| EAEC | 10 | O128:HNT |  |  | **-** | **-** |
| EAEC | 11 | **O104:H4** |  |  | **+** | **-** |
| EAEC | 12 | O86:H2 |  |  | **-** | **-** |
| EAEC | 13 | O17:H18 |  |  | **+** | **+** |
| EAEC | 14 | O11:H18 |  |  | **+** | **+** |
| EAEC | 15 | ONT:H27 |  |  | **-** | **-** |
| EAEC | 101 | O99:H4 |  |  | **-** | **-** |
| ExPEC | 17 | O17:H18 | ST597 | D | **+** | **-** |
| ExPEC | 18 | O7:H18 | ST62 | F | **-** | **-** |
| ExPEC | 19 | O6:H1 | ST73 | B2 | **+** | **+** |
| ExPEC | 20 | O6:H31 | ST127 | B2 | **-** | **-** |
| ExPEC | 21 | O4:H5 | ST12 | B2 | **+** | **-** |
| ExPEC | 22 | O45A:H7 | ST95 | B2 | **-** | **-** |
| ExPEC | 23 | O18:H7 | ST95 | B2 | **-** | **-** |
| ExPEC | 24 | O1:H7 | ST95 | B2 | **-** | **-** |
| ExPEC | 26 | O25b:H4 | ST131 | B2 | **-** | **+** |
| ExPEC | 27 | O25b:H4 | ST131 | B2 | **+** | **+** |
| ExPEC | 28 | O25b:H4 | ST131 | B2 | **+** | **+** |
| ExPEC | 29 | O25b:H4 | ST131 | B2 | **-** | **-** |
| ExPEC | 30 | O25b:H4 | ST131 | B2 | **+** | **+** |
| ExPEC | 31 | O16:H5 | ST131 | B2 | **+** | **-** |
| STEC/VTEC | 32 | O174:H8 |  |  | **+** | **+** |
| STEC/VTEC | 33 | O55:H7 |  |  | **+** | **-** |
| STEC/VTEC | 35 | O177:[H25] |  |  | **-** | **-** |
| STEC/VTEC | 36 | O111:[H8] |  |  | **-** | **-** |
| STEC/VTEC | 37 | O113:H4 |  |  | **+** | **+** |
| STEC/VTEC | 38 | O103:H2 |  |  | **-** | **-** |
| STEC/VTEC | 39 | O26:H11 |  |  | **+** | **+** |
| STEC/VTEC | 40 | O41:H26 |  |  | **-** | **-** |
| STEC/VTEC | 41 | O157:H7 |  |  | **-** | **-** |
| STEC/VTEC | 42 | O157:H7 |  |  | **-** | **-** |
| STEC/VTEC | 43 | O174:H8 |  |  | **+** | **+** |
| STEC/VTEC | 44 | O8:H19 |  |  | **-** | **-** |
| STEC/VTEC | 45 | O48:H21 |  |  | **-** | **-** |
| STEC/VTEC | 46 | O118:H12 |  |  | **+** | **+** |
| STEC/VTEC | 47 | O174:H21 |  |  | **+** | **-** |
| STEC/VTEC | 48 | O73:H18 |  |  | **-** | **+** |
| STEC/VTEC | 49 | O139:H1,H38 |  |  | **+** | **-** |
| STEC/VTEC | 50 | O128:HNM |  |  | **-** | **-** |
| STEC/VTEC | 51 | O2:H25 |  |  | **-** | **-** |
| STEC/VTEC | 55 | O113 |  |  | **+** | **+** |
| STEC/VTEC | 57 | O121:H19 |  |  | **-** | **-** |
| ETEC | 52 | O8:H9 |  |  | **+** | **-** |
| ETEC | 58 | O78:H11 |  |  | **-** | **+** |
| ETEC | 60 | O115:H40 |  |  | **+** | **-** |
| tEPEC | 53 | O26:H11 |  |  | **+** | **+** |
| tEPEC | 34 | O128ac:[H2] |  |  | **-** | **-** |
| EIEC | 56 | O96:H19 |  |  | **-** | **-** |
| EIEC | 59 | O124:H30 |  |  | **-** | **-** |
| AIEC | 25 | O83:H1 | ST135 | B2 | **-** | **-** |
| Comensal | 16 | O16:H48 | ST98 | A1 | **-** | **-** |

|  |  |  |  |
| --- | --- | --- | --- |
| **Pathotype** |  |  |  |
| EAEC | enteroaggregative *E.coli* | |  |
| ExPEC | extraintestinal pathogenic *E.coli* | |  |
| STEC/VTEC | Shiga toxin (verotoxin)-producing *E.coli* |  |  |
| ETEC | enterotoxigenic *E.coli* |  |  |
| tEPEC | typical enteropathogenic *E. coli* |  |  |
| EIEC | enteroinvasive *E.coli* |  |  |
| AIEC | adherent-invasive *E.coli* |  |  |

| Supplementary table 3. Virulende factors and presence of *hha* alleles in the EAEC strains studied. | | | | | | | | | | | | | | | | | | | | | | | | | | | | | | |
| --- | --- | --- | --- | --- | --- | --- | --- | --- | --- | --- | --- | --- | --- | --- | --- | --- | --- | --- | --- | --- | --- | --- | --- | --- | --- | --- | --- | --- | --- | --- |
| **Code**  **LREC** | **Serotype** | **Phylogenetic** | **Virulence genes of enteroaggregative Escherichia coli (EAEC)** | | | | | | | | | | | | | | | | | | | | | | | | |  | | |
| Strain | O:H | group | *aatA* | *aggA* | *aafA* | | *agg3A* | | *aggR* | | *astA* | *aap* | | *set1A* | | *irp2* | | *pet* | | *shf* | | *sat* | | *pic* | | *agn43* | | *hha2* | *hha3* | |
| 66 | O85:HNT | A | **+** | **-** | **-** | | **-** | | **-** | | **-** | **-** | | **-** | | **+** | | **-** | | **+** | | **-** | | **-** | | **-** | | **-** | **-** | |
| 71 | O85:H10 | A | **+** | **-** | **-** | | **-** | | **+** | | **-** | **+** | | **-** | | **+** | | **-** | | **-** | | **-** | | **-** | | **+** | | **-** | **-** | |
| 87 | O3:H2 | A | **+** | **+** | **-** | | **-** | | **+** | | **+** | **+** | | **+** | | **+** | | **+** | | **-** | | **+** | | **+** | | **+** | | **-** | **-** | |
| 95 | O15:H- | A | **+** | **-** | **-** | | **-** | | **+** | | **-** | **+** | | **+** | | **+** | | **+** | | **-** | | **-** | | **+** | | **-** | | **-** | **-** | |
| 82 | O15:H2 | A | **+** | **-** | **-** | | **-** | | **+** | | **-** | **+** | | **+** | | **+** | | **+** | | **-** | | **-** | | **+** | | **-** | | **-** | **-** | |
| 79 | O86:H2 | A | **+** | **-** | **-** | | **+** | | **+** | | **-** | **+** | | **+** | | **+** | | **+** | | **-** | | **-** | | **+** | | **-** | | **-** | **-** | |
| 80 | O86:H2 | A | **+** | **-** | **-** | | **+** | | **+** | | **-** | **+** | | **+** | | **+** | | **+** | | **-** | | **-** | | **+** | | **-** | | **-** | **-** | |
| 89 | O86:H2 | A | **+** | **-** | **-** | | **+** | | **+** | | **-** | **+** | | **+** | | **+** | | **+** | | **-** | | **-** | | **+** | | **-** | | **-** | **-** | |
| 90 | O86:H2 | A | **+** | **-** | **-** | | **+** | | **+** | | **-** | **+** | | **+** | | **+** | | **+** | | **-** | | **-** | | **+** | | **-** | | **-** | **-** | |
| 12 | O86:H2 | A | **+** | **-** | **-** | | **+** | | **+** | | **-** | **+** | | **+** | | **+** | | **+** | | **-** | | **-** | | **+** | | **-** | | **-** | **-** | |
| 101 | O99:H4 | A | **+** | **-** | **-** | | **-** | | **+** | | **-** | **+** | | **+** | | **+** | | **+** | | **-** | | **-** | | **+** | | **-** | | **-** | **-** | |
| 81 | O114:H2 | A | **+** | **-** | **-** | | **+** | | **+** | | **-** | **+** | | **+** | | **+** | | **+** | | **-** | | **-** | | **+** | | **-** | | **-** | **-** | |
| 3 | O11:H- | B1 | **+** | **-** | **+** | | **-** | | **+** | | **+** | **+** | | **+** | | **+** | | **+** | | **+** | | **-** | | **+** | | **+** | | **-** | **-** | |
| 88 | O86:H27 | B1 | **+** | **-** | **-** | | **-** | | **+** | | **-** | **+** | | **+** | | **+** | | **+** | | **+** | | **+** | | **+** | | **+** | | **-** | **-** | |
| 10 | O128:HNT | B1 | **+** | **-** | **-** | | **-** | | **-** | | **-** | **+** | | **-** | | **-** | | **-** | | **-** | | **-** | | **-** | | **-** | | **-** | **-** | |
| 100 | ONT:HNT | B2 | **+** | **-** | **-** | | **-** | | **+** | | **-** | **+** | | **-** | | **+** | | **-** | | **-** | | **-** | | **-** | | **+** | | **-** | **-** | |
| 67 | O86:H11 | D | **+** | **-** | **-** | | **-** | | **+** | | **-** | **+** | | **-** | | **+** | | **-** | | **-** | | **-** | | **-** | | **-** | | **-** | **-** | |
| 83 | O3:H2 | A | **+** | **+** | **-** | | **-** | | **+** | | **+** | **+** | | **+** | | **+** | | **+** | | **-** | | **+** | | **+** | | **+** | | **+** | **-** | |
| 8 | O15:H- | A | **+** | **-** | **-** | | **-** | | **-** | | **-** | **-** | | **+** | | **+** | | **+** | | **-** | | **-** | | **+** | | **-** | | **+** | **-** | |
| 74 | O125:H2 | A | **+** | **-** | **-** | | **-** | | **+** | | **-** | **+** | | **+** | | **+** | | **+** | | **-** | | **-** | | **+** | | **+** | | **+** | **-** | |
| 91 | ONT:H33 | A | **+** | **-** | **-** | | **-** | | **+** | | **-** | **+** | | **+** | | **+** | | **+** | | **-** | | **+** | | **+** | | **+** | | **+** | **-** | |
| 92 | ONT:H33 | A | **+** | **-** | **-** | | **-** | | **+** | | **-** | **+** | | **+** | | **+** | | **+** | | **-** | | **+** | | **+** | | **+** | | **+** | **-** | |
| 96 | ONT:H33 | A | **+** | **-** | **-** | | **-** | | **+** | | **-** | **+** | | **+** | | **+** | | **+** | | **-** | | **+** | | **+** | | **+** | | **+** | **-** | |
| 98 | ONT:H33 | A | **+** | **-** | **-** | | **-** | | **+** | | **-** | **+** | | **+** | | **+** | | **+** | | **-** | | **+** | | **+** | | **+** | | **+** | **-** | |
| 93 | O104:H4 | B1 | **+** | **-** | **-** | | **+** | | **+** | | **+** | **+** | | **+** | | **+** | | **+** | | **+** | | **-** | | **+** | | **-** | | **+** | **-** | |
| 94 | O104:H4 | B1 | **+** | **-** | **-** | | **+** | | **+** | | **+** | **+** | | **+** | | **+** | | **+** | | **+** | | **-** | | **+** | | **+** | | **+** | **-** | |
| 76 | O111:H- | B1 | **+** |  |  | | **-** | | **+** | | **+** | **+** | | **+** | | **+** | | **+** | | **-** | | **+** | | **+** | | **-** | | **+** | **-** | |
| 99 | ONT:HNT | A | **+** | **-** | **-** | | **-** | | **-** | | **+** | **+** | | **-** | | **+** | | **+** | | **-** | | **-** | | **-** | | **-** | | **-** | **+** | |
| 84 | O86:H27 | B1 | **+** | **-** | **-** | | **-** | | **+** | | **-** | **+** | | **+** | | **+** | | **+** | | **+** | | **+** | | **+** | | **+** | | **-** | **+** | |
| 13 | O17:H18 | A | **+** | **-** | **-** | | **-** | | **+** | | **-** | **+** | | **+** | | **-** | | **+** | | **+** | | **-** | | **+** | | **+** | | **+** | **+** | |
| 1 | O3:H2 | A | **+** | **+** | **-** | | **-** | | **+** | | **+** | **+** | | **-** | | **+** | | **-** | | **+** | | **-** | | **-** | | **+** | | **+** | **+** | |
| 2 | O3:H2 | A | **+** | **+** | **-** | | **-** | | **+** | | **+** | **+** | | **-** | | **+** | | **-** | | **-** | | **-** | | **-** | | **+** | | **+** | **+** | |
| 77 | O3:H2 | A | **+** | **+** | **-** | | **-** | | **+** | | **+** | **+** | | **+** | | **+** | | **+** | | **+** | | **+** | | **+** | | **+** | | **+** | **+** | |
| 78 | O3:H2 | A | **+** | **+** | **-** | | **-** | | **+** | | **+** | **+** | | **+** | | **+** | | **+** | | **-** | | **-** | | **+** | | **+** | | **+** | **+** | |
| 7 | O3:H2 | A | **+** | **+** | **-** | | **-** | | **+** | | **+** | **+** | | **+** | | **+** | | **+** | | **-** | | **+** | | **+** | | **+** | | **+** | **+** | |
| 61 | O15:H2 | A | **+** | **-** | **-** | | **+** | | **+** | | **+** | **+** | | **+** | | **+** | | **+** | | **+** | | **+** | | **+** | | **+** | | **+** | **+** | |
| 69 | O78:H2 | A | **+** | **+** | **-** | | **-** | | **+** | | **-** | **+** | | **+** | | **+** | | **+** | | **+** | | **-** | | **+** | | **-** | | **+** | **+** | |
| 70 | O78:H10 | A | **+** | **+** | **-** | | **-** | | **+** | | **+** | **+** | | **+** | | **+** | | **+** | | **-** | | **+** | | **+** | | **-** | | **+** | **+** | |
| 75 | O92:H33 | A | **+** | **-** | **-** | | **-** | | **+** | | **-** | **+** | | **+** | | **+** | | **+** | | **-** | | **+** | | **+** | | **+** | | **+** | **+** | |
| 62 | O168:H4 | A | **+** | **+** | **-** | | **-** | | **+** | | **+** | **+** | | **-** | | **-** | | **+** | | **-** | | **+** | | **-** | | **-** | | **+** | **+** | |
| 85 | O168:H4 | A | **+** | **+** | **-** | | **-** | | **+** | | **+** | **+** | | **-** | | **-** | | **+** | | **-** | | **+** | | **-** | | **-** | | **+** | **+** | |
| 68 | O61:H4 | B1 | **+** | **-** | **-** | | **-** | | **+** | | **-** | **+** | | **+** | | **+** | | **+** | | **+** | | **+** | | **+** | | **+** | | **+** | **+** | |
| 11 | O104:H4 | B1 | **+** | **-** | **-** | | **+** | | **+** | | **+** | **+** | | **+** | | **+** | | **+** | | **+** | | **-** | | **+** | | **+** | | **+** | **+** | |
| 72 | ONT:H28 | B1 | **+** | **-** | **+** | | **-** | | **+** | | **+** | **+** | | **+** | | **+** | | **+** | | **+** | | **-** | | **+** | | **+** | | **+** | **+** | |
| 73 | ONT:H28 | B1 | **+** | **-** | **+** | | **-** | | **+** | | **+** | **+** | | **+** | | **+** | | **+** | | **+** | | **-** | | **+** | | **+** | | **+** | **+** | |
| 6 | ONT:HNT | B1 | **+** | **-** | **+** | | **-** | | **+** | | **+** | **+** | | **+** | | **-** | | **+** | | **+** | | **-** | | **+** | | **+** | | **+** | **+** | |
| 63 | O17:H2 | B2 | **+** | **+** | **-** | | **-** | | **+** | | **-** | **+** | | **+** | | **+** | | **+** | | **+** | | **+** | | **+** | | **+** | | **+** | **+** | |
| 14 | O11:H18 | D | **+** | **+** | **-** | | **-** | | **+** | | **-** | **+** | | **+** | | **+** | | **+** | | **+** | | **+** | | **+** | | **+** | | **+** | **+** | |
| 64 | O15:H18 | D | **+** | **-** | **-** | | **+** | | **+** | | **-** | **+** | | **+** | | **+** | | **+** | | **-** | | **+** | | **+** | | **+** | | **+** | **+** | |
| 65 | O15:H- | D | **+** | **-** | **-** | | **+** | | **+** | | **-** | **+** | | **+** | | **+** | | **+** | | **+** | | **+** | | **+** | | **+** | | **+** | **+** | |
| 97 | O15:H1 | D | **+** | **-** | **-** | | **-** | | **-** | | **-** | **-** | | **-** | | **+** | | **+** | | **-** | | **+** | | **-** | | **+** | | **+** | **+** | |
| 4 | O17:H- | D | **+** | **-** | **+** | | **-** | | **+** | | **+** | **+** | | **+** | | **+** | | **+** | | **+** | | **-** | | **+** | | **+** | | **+** | **+** | |
| 9 | O17:H18 | D | **+** | **-** | **-** | | **-** | | **+** | | **-** | **+** | | **-** | | **-** | | **-** | | **+** | | **-** | | **-** | | **+** | | **+** | **+** | |
| 5 | O44:H18 | D | **+** | **-** | **+** | | **-** | | **+** | | **+** | **+** | | **+** | | **+** | | **+** | | **+** | | **-** | | **+** | | **+** | | **+** | **+** | |
| 86 | O73-44:H18 | D | **+** | **-** | **-** | | **-** | | **+** | | **-** | **+** | | **+** | | **-** | | **+** | | **+** | | **-** | | **+** | | **+** | | **+** | **+** | |
| 15 | ONT:H27 | D | **+** | **-** | **-** | | **-** | | **-** | | **-** | **-** | | **+** | | **+** | | **+** | | **-** | | **+** | | **+** | | **+** | | **+** | **+** | |
| Code LREC | Serotype | Phylogenetic | Virulence genes of enteroaggregative Escherichia coli (EAEC) | | | | | | | | | | | | | | | | | | | | | | | | |  | | |
| Strain | O:H | group | *aatA* | *aggA* | | *aafA* | | *agg3A* | *aggR* | *astA* | | | *aap* | | *set1A* | | *irp2* | | *pet* | | *shf* | | *sat* | | *pic* | | *agn43* | *hha*  *2* | | *hha3* |
|  |  | TOTAL | 56 | 13  23% | | 6 11% | | 12  21% | 50 89% | 22 39% | | | 52 93% | | 44 79% | | 49 88% | | 48 86% | | 23 41% | | 23  41% | | 44 79% | | 36  64% | 37 | | 29 |
|  |  | hha2-/hha3- | 17 | **1**  **6%** | | 1  6% | | 6  35% | 15 88% | **2**  **12%** | | | 16 94% | | 12 71% | | 16 94% | | 12 71% | | **3**  **18%** | | **2**  **12%** | | 12 71% | | **5**  **29%** |  | |  |
|  |  | hha2+/hha3- | 10 | **1**  **10%** | | 0 | | 2  20% | 9 90% | **4**  **40%** | | | 9  90% | | 10 100% | | 10 100% | | 10 100% | | **2**  **20%** | | **6**  **60%** | | 10 100% | | **7**  **70%** |  | |  |
|  |  | hha2-/hha3+ | 2 | **0** | | 0 | | 0 | 1 50% | **1**  **50%** | | | 2 100% | | 1 50% | | 2 100% | | 2 100% | | **1**  **50%** | | **1**  **50%** | | 1 50% | | **1**  **50%** |  | |  |
|  |  | hha2+/hha3+ | 27 | **11**  **41%** | | 5 19% | | 4 15% | 25 93% | **15 56%** | | | 25 93% | | 21 78% | | 21 78% | | 24 89% | | **17 63%** | | **14**  **52%** | | 21 78% | | **23**  **85%** |  | |  |
| Fisher´s exact test | P value | hha2-/hha3- vs hha2+/hha3+ |  | **0.01** | |  | |  |  | **0.004** | | |  | |  | |  | |  | | **0.004** | | **0.007** | |  | | **0.0003** |  | |  |
|  |  |  |  |  | |  | |  |  |  | | |  | |  | |  | |  | |  | |  | |  | |  |  | |  |
|  |  |  |  |  | |  | |  |  |  | | |  | |  | |  | |  | |  | |  | |  | |  |  | |  |
| Fisher´s exact test | P value | hha2-/hha3- vs hha2+ and/or hha3+ |  | **0.04** | |  | |  |  | **0.005** | | |  | |  | |  | |  | | **0.02** | | **0.003** | |  | | **0.0005** |  | |  |
| Fisher´s exact test are shown where P < 0.05. Significant differences are indicated in bold | | | | | | | | | | | | | | | | | | | | | | | | | | | |  | |  |

| **Gene** | Oligonucleotides 5´- 3´ | | Amplicon size (bp) | Reference |  |
| --- | --- | --- | --- | --- | --- |
| *aggR* | aggR-F | CTAATTGTACAATCGATGTA | 308 | **1** |  |
|  | aggR-R | ATGAAGTAATTCTTGAAT |  |  |  |
| *aggA* | aggA-F | TTAGTCTTCTATCTAGGG | 450 | **1** |  |
|  | aggA-R | AAATTAATTCCGGCATGG |  |  |  |
| *aafA* | aafA-F | ATGTATTTTTAGAGGTTGAC | 518 | **1** |  |
|  | aafA-R | TATTATATTGTCACAAGCTC |  |  |  |
| *aap* | aap-F | CTTTTCTGGCATCTTGGGT | 232 | **2** |  |
|  | aap-R | GTAACAACCCCTTTGGAAGT |  |  |  |
| *set1A* | set1A-F | TCACGCTACCATCAAAGA | 309 | **3** |  |
|  | set1A-R | TATCCCCCTTTGGTGGTA |  |  |  |
| *astA* | astA-F | CCATCAACACAGTATATCCGA | 111 | **4** |  |
|  | astA-R | GGTCGCGAGTGACGGCTTTGT |  |  |  |
| *irp2* | irp2-F | AAGGATTCGCTGTTACCGGAC | 264 | **1** |  |
|  | irp2-R | TCGTCGGGCAGCGTTTCTTCT |  |  |  |
| *pet* | pet-F | GACCATGACCTATACCGACAGC | 599 | **1** |  |
|  | pet-R | CCGATTTCTCAAACTCAAGACC |  |  |  |
| *shf* | shf-F | ACTTTCTCCCGAGACATTC | 613 | **1** |  |
|  | shf-R | CTTTAGCGGGAGCATTCAT |  |  |  |
| *agg-3A* | agg-3A-F | GTATCATTGCGAGTCTGGTATTCAG | 462 | **1** |  |
|  | agg-3A-R | GGGCTGTTATAGAGTAACTTCCAG |  |  |  |
| *aatA* | aat-F | CTGGCGAAAGACTGTATCAT | 629 | **5** |  |
|  | aat-R | AATGTATAGAAATCCGCTGTT |  |  |  |
| *agn43* | agn43-F | ACGCACAACCATCAATAAAA | 600 | **5** |  |
|  | agn43-R | CCGCCTCCGATACTGAATGC |  |  |  |
| *sat* | sat-F | ACTGGCGGACTCATGCTGT | 387 | **5** |  |
|  | sat-R | AACCCTGTAAGAAGACTGAGC |  |  |  |
| *pic* | pic-F | TTCAGCGGAAAGACGAA | 517 | **6** |  |
|  | pic-R | TCTGCGCATTCATACCA |  |  |  |
|  |  |  |  |  |  |

|  |  |
| --- | --- |
| Virulence genes | Virulence factos |
| *aatA* | Antiaggregation protein transporter |
| *aggA* | AAF/I fimbrial subunit |
| *aafA* | AAF/II fimbrial subunit |
| *agg3A* | AAF/III fimbrial subunit |
| *aggR* | Transcriptonal activator |
| *astA* | Aggregative heat-stable toxin 1 (EAST1) |
| *aap* | Anti-aggregation protein (Dispersin) |
| *set1A* | *Shigella* enterotoxin 1 mucinase |
| *irp2* | *Yersinia* bactin biosynthesis gene |
| *pet* | Plasmid-encoded toxin (serine protease) |
| *shf* | Cryptic ORF |
| *sat* | Secreted autotransporter toxin |
| *pic* | Serine protease Pic |
| *agn43* | Antigen 43 |
|  |  |

1. Czeczulin, J. R., Whittam, T. S., Henderson, I. R., Navarro-Garcia, F. & Nataro, J. P. Phylogenetic analysis of enteroaggregative and diffusely adherent Escherichia coli. *Infect. Immun.* **67,** 2692–2699 (1999).

2. Sheikh, J. *et al.* A novel dispersin protein in enteroaggregative Escherichia coli. *J. Clin. Invest.* **110,** 1329–1337 (2002).

3. Fasano, A. *et al.* Shigella enterotoxin 1: an enterotoxin of Shigella flexneri 2a active in rabbit small intestine in vivo and in vitro. *J. Clin. Invest.* **95,** 2853–2861 (1995).

4. Yatsuyanagi, J., Saito, S., Miyajima, Y., Amano, K.-I. & Enomoto, K. Characterization of atypical enteropathogenic Escherichia coli strains harboring the astA gene that were associated with a waterborne outbreak of diarrhea in Japan. *J. Clin. Microbiol.* **41,** 2033–2039 (2003).

5. Mendez-Arancibia, E. *et al.* Prevalence of different virulence factors and biofilm production in enteroaggregative Escherichia coli isolates causing diarrhea in children in Ifakara (Tanzania). *Am. J. Trop. Med. Hyg.* **78,** 985–989 (2008).

6. Piva, I. C. *et al.* Virulence markers of enteroaggregative Escherichia coli isolated from children and adults with diarrhea in Brasília, Brazil. *J. Clin. Microbiol.* **41,** 1827–1832 (2003).

| Supplementary Table 4 - Collection of 25 *E. coli* isolates from German humans and companion animals. | | | | | | | | |
| --- | --- | --- | --- | --- | --- | --- | --- | --- |
| **Isolate** | **source** | **material** | **ESBL gene** | **Pathotype** | **phylogenetic group** | **sequence type** | ***hha2*** | ***hha3*** |
| H10 | out-patient | urine | CTX-M-15 | NA^*^ | B1 | ST-443 | **+** | **+** |
| H38 | in-patient | rectal swab | CTX-M-15 | ExPEC | A | ST-44 | **-** | **+** |
| H75 | in-patient | urine | CTX-M-15 | ExPEC | B2 | ST-131 | **+** | **+** |
| H89 | in-patient | wound swab | CTX-M-15 | NA* | D | ST-349 | **-** | **+** |
| H99 | out-patient | wound swab | CTX-M-1 | ExPEC | A | ST-410 | **-** | **-** |
| H127 | in-patient | urine | CTX-M-15 | EAEC | B2 | ST-131 | **+** | **+** |
| H130 | out-patient | cervical swab | CTX-M-15 | ExPEC | A | ST-88 | **+** | **+** |
| H134 | in-patient | urine | CTX-M-15 | ExPEC | B1 | ST-58 | **-** | **+** |
| H136 | out-patient | urine | CTX-M-1 | ExPEC | D | ST-2509 | **-** | **-** |
| H141 | out-patient | urine | CTX-M-15 | ExPEC | D | ST-2141 | **-** | **+** |
| H154 | in-patient | rectal swab | CTX-M-15 | Intestinal flora | B1 | ST-224 | **-** | **-** |
| H162 | out-patient | urine | CTX-M-1 | NA^*^ | A | ST-10 | **+** | **-** |
| V1 | dog | urine | CTX-M-1 | EAEC | D | ST-354 | **-** | **+** |
| V8 | horse | faeces | CTX-M-1 | Intestinal flora | D | ST-38 | **-** | **-** |
| V41 | dog | abdominal cavity | CTX-M-1 | ExPEC | A | ST-10 | **+** | **+** |
| V63 | dog | content of uterus | CTX-M-1 | ExPEC | B1 | ST-162 | **-** | **-** |
| V64 | dog | content of uterus | CTX-M-1 | ExPEC | B1 | ST-162 | **-** | **-** |
| V89 | horse | faeces | CTX-M-1 | Intestinal flora | A | ST-10 | **-** | **-** |
| V98 | horse | faeces | CTX-M-15 | Intestinal flora | A | ST-361 | **-** | **+** |
| V105 | dog | urine | CTX-M-1 | ExPEC | A | ST-3476 | **-** | **+** |
| V116 | dog | wound swab | CTX-M-1 | NA* | A | ST-10 | **-** | **-** |
| V177 | dog | liver | CTX-M-15 | ExPEC | A | ST-410 | **+** | **+** |
| V182 | horse | faeces | CTX-M-15 | Intestinal flora | A | ST-10 | **-** | **+** |
| V205 | cat | urine | CTX-M-1 | ExPEC | B1 | ST-224 | **-** | **-** |
| V292 | dog | catheter urine | CTX-M-15 | ExPEC | D | ST-405 | **+** | **-** |
|  |  |  |  |  |  |  |  |  |
| ^*^No pathotype could be assigned | | |  |  |  |  |  |  |

Supplementary Table 5: Comparison of PCR results for detection of hha homologues compared with *in silico* results. *In silico* identification of hha homologues was performed using BLASTN with a cutoff of 90% identity and 80% coverage.

|  |  | **EC042_4516** | | **EC042_4796** | |
| --- | --- | --- | --- | --- | --- |
| **Isolate** | **Sequence type** | **PCR result** | **In silico** | **PCR result** | **In silico** |
| **H10** | **443** | **-** | **+** | **+** | **+** |
| **H38** | **44** | **-** | **-** | **+** | **+** |
| **H75** | **131** | **-** | **-** | **+** | **+#** |
| **H89** | **349** | **-** | **-** | **+** | **+** |
| **H99** | **410** | **-** | **-** | **-** | **-** |
| **H127** | **131** | **+** | **+** | **+** | **+#** |
| **H130** | **131** | **+** | **+** | **+** | **+#** |
| **H134** | **58** | **-** | **-** | **+** | **+** |
| **H136** | **2509** | **-** | **-** | **-** | **-** |
| **H141** | **2141** | **-** | **-** | **+** | **+** |
| **H154** | **224** | **-** | **-** | **-** | **-** |
| **H162** | **10** | **+** | **+** | **-** | **-** |
| **V1** | **354** | **-** | **-** | **+** | **+** |
| **V41** | **10** | **+** | **+*** | **+** | **+*** |
| **V63** | **162** | **-** | **+** | **-** | **-** |
| **V64** | **162** | **-** | **+** | **-** | **-** |
| **V8** | **38** | **-** | **-** | **-** | **-** |
| **V89** | **10** | **-** | **-** | **-** | **-** |
| **V98** | **361** | **-** | **-** | **+** | **+** |
| **V105** | **3476** | **-** | **-** | **+** | **+** |
| **V116** | **10** | **-** | **-** | **-** | **-** |
| **V177** | **410** | **+** | **+** | **+** | **+** |
| **V182** | **10** |  |  | **+** | **+** |
| **V205** | **224** |  |  |  |  |
| **V292** | **405** | **+** | **+** |  |  |

*these homologues are located at the same position in the genome; # two homologues with different identity/coverage present

| Supplementary Table 6. Features of the strains belonging to the EcoR collection and distribution of *hha2* and *hha3* alleles. | | | | | | | | | | | |
| --- | --- | --- | --- | --- | --- | --- | --- | --- | --- | --- | --- |
|  | | | | | | | | | | | |
| **Strain** | **Pathotype** | ***hha2*** | ***hha3*** | **Strain** | **Pathogenic category** | ***hha2*** | ***hha3*** | **Strain** | **Pathogenic category** | ***hha2*** | ***hha3*** |
| **EC01** | Commensal | **-** | **-** | **EC26** | Commensal | **-** | **-** | **EC51** | ExPEC | **+** | **+** |
| **EC02** | ExPEC | **-** | **-** | **EC27** | Commensal | **-** | **-** | **EC52** | ExPEC | **-** | **-** |
| **EC03** | Commensal | **-** | **+** | **EC28** | Commensal | **-** | **-** | **EC53** | ExPEC | **-** | **-** |
| **EC04** | Commensal | **-** | **-** | **EC29** | Commensal | **-** | **-** | **EC54** | ExPEC | **-** | **-** |
| **EC05** | Commensal | **+** | **-** | **EC30** | Commensal | **-** | **-** | **EC55** | ExPEC | **-** | **+** |
| **EC06** | Commensal | **-** | **-** | **EC31** | Commensal | **-** | **-** | **EC56** | ExPEC | **-** | **+** |
| **EC07** | Commensal | **-** | **-** | **EC32** | Commensal | **-** | **-** | **EC57** | ExPEC | **+** | **-** |
| **EC08** | ExPEC | **+** | **-** | **EC33** | Commensal | **-** | **-** | **EC58** | Commensal | **-** | **-** |
| **EC09** | Commensal | **-** | **+** | **EC34** | Commensal | **-** | **-** | **EC59** | Commensal | **+** | **-** |
| **EC10** | Commensal | **-** | **+** | **EC35** | ExPEC | **-** | **-** | **EC60** | ExPEC | **+** | **+** |
| **EC11** | ExPEC | **+** | **-** | **EC36** | ExPEC | **-** | **-** | **EC61** | Commensal | **-** | **-** |
| **EC12** | Commensal | **-** | **-** | **EC37** | ExPEC | **-** | **+** | **EC62** | ExPEC | **-** | **-** |
| **EC13** | Commensal | **-** | **-** | **EC38** | ExPEC | **-** | **-** | **EC63** | ExPEC | **-** | **-** |
| **EC14** | Commensal | **-** | **-** | **EC39** | ExPEC | **-** | **-** | **EC64** | ExPEC | **-** | **-** |
| **EC15** | Commensal | **-** | **-** | **EC40** | ExPEC | **-** | **-** | **EC65** | Commensal | **+** | **-** |
| **EC16** | Commensal | **-** | **+** | **EC41** | ExPEC | **-** | **-** | **EC66** | ExPEC | **-** | **-** |
| **EC17** | Commensal | **-** | **-** | **EC42** | Commensal | **-** | **-** | **EC67** | Commensal | **-** | **-** |
| **EC18** | Commensal | **-** | **-** | **EC43** | Commensal | **+** | **-** | **EC68** | Commensal | **-** | **-** |
| **EC19** | Commensal | **-** | **-** | **EC44** | Commensal | **+** | **-** | **EC69** | Commensal | **-** | **-** |
| **EC20** | ExPEC | **-** | **-** | **EC45** | Commensal | **-** | **-** | **EC70** | Commensal | **-** | **-** |
| **EC21** | Commensal | **-** | **-** | **EC46** | ExPEC | **-** | **-** | **EC71** | Commensal | **-** | **-** |
| **EC22** | Commensal | **-** | **-** | **EC47** | Commensal | **-** | **-** | **EC72** | ExPEC | **-** | **+** |
| **EC23** | Commensal | **-** | **-** | **EC48** | ExPEC | **-** | **-** |  |  |  |  |
| **EC24** | ExPEC | **-** | **-** | **EC49** | ExPEC | **-** | **-** |  |  |  |  |
| **EC25** | Commensal | **-** | **-** | **EC50** | ExPEC | **+** | **+** |  |  |  |  |

| Supplementary Table 7**-** Environmental *stx*_2_ positive isolates from wastewater of different animal origins. | | | | | | | |
| --- | --- | --- | --- | --- | --- | --- | --- |
| **Isolate** | **Origin** | **Serotype** | ***stx_1_*** | ***stx_2_*** | ***eae*** | ***hha2*** | ***hha3*** |
| 41 (115) | Cattle | O1:H20 | **-** | *stx2a* | **-** | **-** | **-** |
| 57 stx2-r | Cattle | O171:H2 | **-** | *stx2c* | **-** | **-** | **-** |
| 58 stx2-r | Cattle | O171:H2 | **-** | *stx2c* | **-** | **+** | **-** |
| 58 stx2 TAR 43 | Cattle | O101:H9 | **-** | *stx2a* | **-** | **-** | **-** |
| 59 stx2ME-k2 | Cattle | O171:H2 | **-** | *stx2c* | **-** | **+** | **-** |
| 60 stx2-r | Cattle | O171:H2 | **-** | *stx2a* | **-** | **+** | **+** |
| 62 stx2-r | Cattle | O171:H2 | **-** | *stx2c* | **-** | **+** | **+** |
| 63 stx2-r | Cattle | O171:H2 | **-** | *stx2c* | **-** | **+** | **-** |
| 64 stx2-r | Cattle | O171:H2 | **-** | *stx2c* | **-** | **-** | **-** |
| 65 stx2-r | Cattle | O171:H2 | **-** | *stx2a* | **-** | **-** | **-** |
| 66 stx2-ME-8 | Cattle | O181:H49 | **-** | *stx2* | **-** | **-** | **-** |
| 67 stx2-r | Cattle | O171:H2 | **-** | *stx2c* | **-** | **+** | **-** |
| 68 stx2-r | Cattle | O89:H19 | **-** | *stx2c* | **-** | **-** | **-** |
| 69 stx2-r | Cattle | O181:H49 | **-** | *stx2a* | **-** | **+** | **-** |
| 70 stx2 ME-16 | Cattle | O162:H7 | **-** | *stx2, stx2c* | **-** | **-** | **-** |
| 71 stx2-r | Cattle | O28:H28 | **-** | *stx2c* | **+** | **-** | **-** |
| Ex E72 | Cattle | O181:H20 | **-** | *Stx2a* | **-** | **+** | **-** |
| 72 stx2-r | Cattle | O181:H20 | **-** | *stx2c* | **-** | **+** | **-** |
| 73 stx2-r | Cattle | O181:H20 | **-** | *stx2c* | **-** | **+** | **+** |
| 74 stx2-r | Cattle | O171:H2 | **-** | *stx2c* | **-** | **+** | **+** |
| 82 stx2-r | Cattle | O171:H2 | **-** | *stx2c* | **-** | **+** | **-** |
| 83 stx2-r | Cattle | O2:H25 | **-** | *stx2a* | **-** | **+** | **-** |
| 84 stx2-r | Cattle | O156:H8 | **-** | *stx2c* | **-** | **-** | **-** |
| 84b stx2-r | Cattle | O171:H- | **-** | *stx2a* | **-** | **-** | **-** |
| 85 stx2-r | Cattle | O171:H2 | **-** | *stx2c* | **-** | **+** | **-** |
| 86 stx2-r | Cattle | O2:H25 | **-** | *stx2g* | **-** | **-** | **-** |
| 87 stx2-r | Cattle | O2:H25 | **-** | *tx2a* | **-** | **+** | **-** |
| 88 stx2-r | Cattle | O136:H1,H12 | **-** | *stx2g* | **-** | **-** | **-** |
| *Ec* 88 | Cattle | O157:H7 | **-** | *stx2a* | **+** | **-** | **-** |
| 89 stx2-r | Cattle | O2:H25 | **-** | *stx2g* | **-** | **-** | **-** |
| 90 stx2-r | Cattle | O2:H25 | **-** | *stx2g* | **-** | **+** | **-** |
| 91 stx2-r | Cattle | O2:H25 | **-** | *stx2g* | **-** | **-** | **-** |
| 92 stx2-r | Cattle | O2:H25 | **-** | *stx2g* | **-** | **-** | **-** |
| 93 stx2-r | Cattle | O2:H25 | **-** | *stx2g* | **-** | **+** | **-** |
| 95 stx2-r | Cattle | O2:H25 | **-** | *stx2g* | **-** | **-** | **-** |
| 96 stx2 ME | Cattle | ONT:H- | **-** | *stx2c* | **-** | **-** | **-** |
| 98 stx2-r | Cattle | O136:H1 | **-** | *stx2g* | **-** | **+** | **-** |
| 99 stx2-r | Cattle | ONT:HNT | **-** | *stx2c* | **-** | **-** | **-** |
| 100 stx2-r | Cattle | O2:H25 | **-** | *Stx2a* | **-** | **+** | **-** |
| 101 stx2-r | Cattle | O157:H7 | **-** | *stx2c* | **+** | **-** | **-** |
| 102 stx2 ME | Cattle | O8:H31 | **-** | *stx2g* | **-** | **+** | **-** |
| 103 stx2-r | Cattle | O171:H2 | **-** | *stx2a* | **-** | **-** | **-** |
| 104 stx2-r | Cattle | O113:H21 | **-** | *stx2a* | **-** | **-** | **-** |
| 105 stx2 ME | Cattle | O157:H7 | **-** | *stx2c* | **+** | **-** | **-** |
| 106 stx2 ME | Cattle | O171:H2 | **-** | *stx2a, stx2c* | **-** | **+** | **-** |
| 107 stx2-r | Cattle | O2:H25 | **-** | *stx2g* | **-** | **-** | **-** |
| 108 stx2-r | Cattle | O171:H2 | **-** | *stx2c* | **-** | **+** | **-** |
| 109 stx2-r | Cattle | O113:H21 | **-** | *stx2a* | **-** | **+** | **-** |
| 110 stx-2 ME | Cattle | O157:H- | **+** | *stx2c* | **+** | **-** | **-** |
| 111 stx2 ME | Cattle | O157:H- | **-** | *stx2c* | **+** | **+** | **-** |
| 113 stx2-r | Cattle | O171:H2 | **-** | *stx2c* | **-** | **-** | **-** |
| 115 stx2-r | Cattle | O91:H21 | **-** | *stx2a, stx2c* | **-** | **-** | **-** |
| 116 stx2-r | Cattle | O76:H2 | **+** | *stx2c* | **-** | **+** | **-** |
| 124 | Cattle | ONT:H2 | **-** | *stx2c,stx2d* | **-** | **+** | **-** |
| 125-14 | Cattle | O1:H20 | **-** | *stx2a* | **-** | **-** | **-** |
| 126-2 | Cattle | O22:H8 | **+** | *stx2c* | **-** | **+** | **-** |
| O3 | Cattle | O1:H20 | **-** | *stx2a* | **-** | **-** | **-** |
| 4 | Human | O90:H- | **-** | *stx2d* | **-** | **+** | **-** |
| 7 | Human | O8:H9 | **-** | *stx2e* | **-** | **+** | **-** |
| 8 stx2-r | Human | O146:H- | **-** | *stx2a* | **-** | **-** | **-** |
| 51 stx2-r | Human | O146:H21 | **+** | *stx2d* | **-** | **+** | **+** |
| 52 stx2-r | Human | O171:H2 | **-** | *stx2c, stx2d* | **-** | **-** | **-** |
| 55 stx2-r | Human | O127:H- | **-** | *stx2a, stx2d* | **-** | **+** | **-** |
| 77 stx2-r | Human | O90:H- | **-** | *stx2d* | **-** | **+** | **-** |
| 78 stx2-r | Human | O166:H21 | **+** | *stx2c* | **-** | **+** | **+** |
| 79 stx2-r | Human | O90:H- | **+** | *stx2d* | **-** | **+** | **-** |
| 80 stx2-r | Human | ONT:H9 | **-** | *stx2a, stx2e* | **-** | **-** | **-** |
| 81 stx2-r | Human | ONT:H2 | **-** | *stx2a* | **-** | **+** | **-** |
| 118-2 | Human | ONT:H51 | **-** | *stx2a* | **-** | **-** | **-** |
| 121 | Human | O8:H9 | **-** | *stx2e* | **-** | **-** | **-** |
| 122-12 stx2 | Human | O8:H9 | **-** | *stx2a* | **-** | **+** | **-** |
| 42 stx2-r | Mixed | ONT:H48 | **-** | *stx2a* | **-** | **-** | **-** |
| 49 stx2-r | Mixed | O26:H- | **-** | *stx2a, stx2e* | **-** | **-** | **-** |
| 133stx2-r | Mixed | O90:H- | **+** | *stx2d* | **-** | **+** | **-** |
| 48 stx2-r | Mixed | ONT:H21 | **-** | *stx2e* | **-** | **+** | **-** |
| 134 stx2-r | Mixed | O2:H25 | **-** | *stx2g* | **-** | **-** | **-** |
| 135 stx2 Fr2 12 | Mixed | O9:H- | **-** | *stx2a* | **-** | **-** | **-** |
| 140 stx2 Fr3 26 | Mixed | ONT:H- | **-** | *stx2e* | **-** | **+** | **-** |
| 141 stx2 Fr3 29 | Mixed | ONT:H- | **-** | *stx2e* | **-** | **-** | **-** |
| 75 stx2-r | Pig | O2:H21 | **-** | *stx2e* | **-** | **+** | **-** |
| 76 stx2-r | Pig | O54:H21 | **-** | *stx2e* | **-** | **-** | **-** |
| 148 stx2 Fr1.20 | Pig | O100:H- | **-** | *stx2e* | **-** | **-** | **-** |
| 149a stx2 | Pig | O8:H- | **-** | *stx2e* | **-** | **+** | **-** |
| 150 stx2-r | Pig | O26:H- | **-** | *stx2e* | **-** | **-** | **-** |

**Supplementary figures**

**

**

Supplementary Figure S1. Binding sites of the PCR primers used for the amplification the *hha*, *ydgT*, *hha2* and *hha3* genes. Arrows overlap with the oligonucleotide sequence to which the corresponding primers hybridize. The length of each amplicon is indicated in the corresponding box.


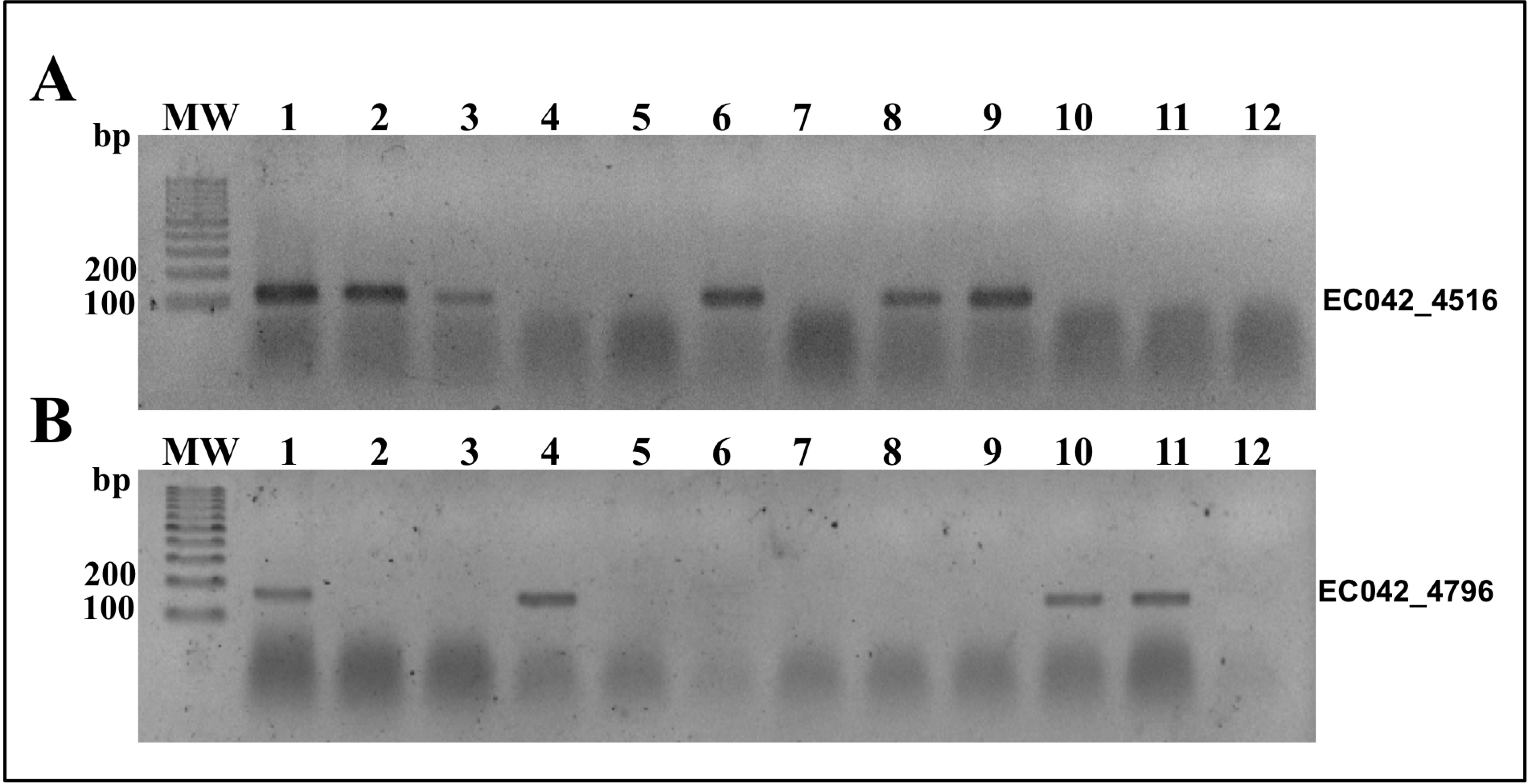


Supplementary Figure S2. (A) PCR amplification of the *hha2* gene in different strains from 84 stx2-positive environmental isolates collection. MW, molecular weight marker (GeneRuler 100 bp DNA Ladder, ThermoFisher Scientific); Lane 1, strain 042; lane 2, strain 62 stx2-r; lane 3, strain 63 stx2-r; lane 4, strain 64 stx2-r; lane 5, strain 65 stx2-r; lane 6, strain 67 stx2-r; lane 7, strain 68 stx2-r; lane 8, strain 108 stx2-r; lane 9, strain 109 stx2-r; lane 10, strain 113 stx2-r; lane 11, strain 115 stx2-r; lane 12, negative PCR control. (B) PCR amplification of the *hha3* gene in different strains from ECOR collection. MW, molecular weight marker (GeneRuler 100 bp DNA Ladder, ThermoFisher Scientific); Lane 1, strain 042; lane 2, strain 1; lane 3, strain 2; lane 4, strain 3; lane 5, strain 4; lane 6, strain 5; lane 7, strain 6; lane 8, strain 7; lane 9, strain 8; lane 10, strain 9; lane 11, strain 10; lane 12, negative PCR control.


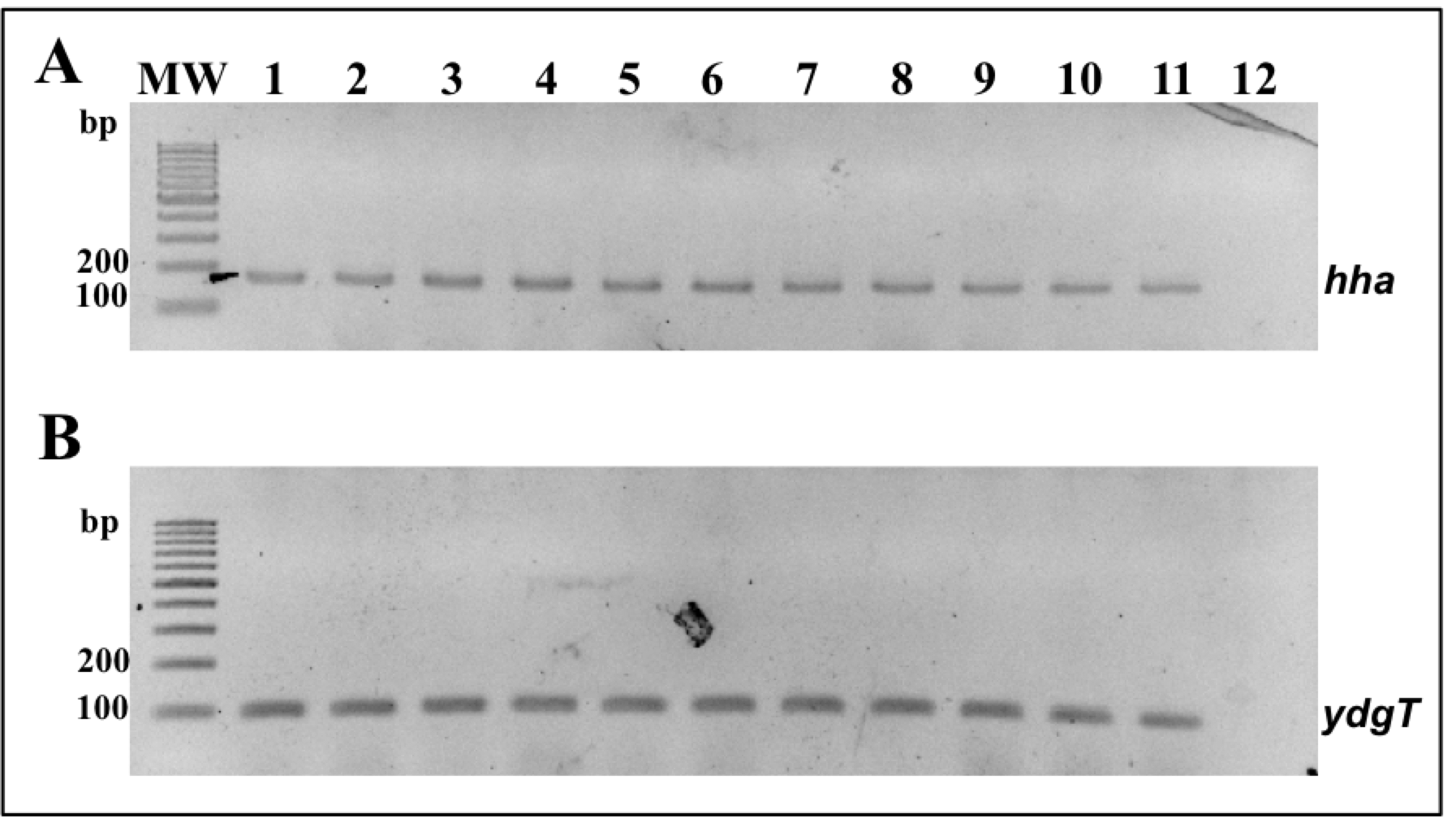


Supplementary Figure S3. PCR amplification of the *hha* (A) and *ydgT* (B) genes in different strains from LREC collection that encode *hha*, *hha2* and *hha3* genes. MW, molecular weight marker (GeneRuler 100 bp DNA Ladder, ThermoFisher Scientific); Lane 1, strain 042; lane 2, strain LREC 3; lane 3, strain LREC 4; lane 4, strain LREC 17; lane 5, strain LREC 24; lane 6, strain LREC 25; lane 7, strain LREC 34; lane 8, strain LREC 35; lane 9, strain LREC 52; lane 10, strain LREC 56; lane 11, strain LREC 60; lane 12, negative PCR control.
